# Evaluation of translational potential of mRNA vaccine candidate antigens for pancreatic cancer: a systematic review based on clinical evidence and stratified prioritization strategies

**DOI:** 10.3389/fimmu.2026.1803792

**Published:** 2026-04-28

**Authors:** Yuzhe Xue, Jiaqi Yu, Hongkun Zhou, Wei Chen, Qi Chen, Lingyu Hu, Runzhou Luo, Yingjing Chen, Xiaoguang Wang, Xuesong He

**Affiliations:** 1 Jiaxing University Master Degree Cultivation Base, Zhejiang Chinese Medical University, Hangzhou, Zhejiang, China; 2 Department of Hepatobiliary Surgery, The Second Affiliated Hospital of Jiaxing University, Jiaxing, Zhejiang, China; 3 Department of Hepatobiliary Surgery, The First Affiliated Hospital of Jiaxing University, Jiaxing, Zhejiang, China; 4 Department of General Surgery, Traditional Chinese Medical Hospital of Zhuji, Zhuji, Zhejiang, China

**Keywords:** antigen selection, antigen stratification, mRNA vaccine, pancreatic ductal adenocarcinoma, tumor immunogenicity

## Abstract

Pancreatic cancer is a highly aggressive malignancy with a 5-year relative survival rate of only 13%. Current treatment options have limited efficacy, and mRNA vaccines offer a new direction for its treatment. However, how to accurately identify antigen targets that possess tumor specificity, functional relevance, and immunogenicity remains the key bottleneck restricting the clinical translation of mRNA vaccines for pancreatic cancer. Recent clinical studies have advanced KRAS mutant vaccines and personalized neoantigen mRNA vaccines, yet most rely on single antigens or highly individualized designs, limiting scalability and broader clinical applicability. In this systematic review, we integrated evidence from public databases and experimental studies to identify and evaluate 16 potential pancreatic cancer mRNA vaccine antigens (ADAM9, WNT7A, TMOD3, MET, EFNB2, TPX2, AGPS, OSBPL9, KDM5A, NRAS, SCP-1, GAGE, RAB5A, ANO6, CHMP2B, and PAK2). All candidates were initially selected based on aberrant tumor expression and further prioritized using stratification strategies incorporating antigen-presenting cell infiltration, immune-related cell death pathways such as ferroptosis and pyroptosis, and functional relevance to tumor progression. ADAM9 and PAK2 showed high expression in pancreatic cancer and strong associations with tumor proliferation, invasion, and immune regulation. SCP-1 and GAGE, as cancer–testis antigens, exhibited high tumor specificity and immunogenic potential. In addition, KDM5A and ANO6 may enhance antitumor efficacy through modulation of ferroptosis or pyroptosis. Nevertheless, several candidates remain constrained by normal tissue expression or limited mechanistic evidence. This review provides a stratified framework for antigen prioritization and highlights key challenges in pancreatic cancer mRNA vaccine development, offering guidance for future multi-antigen vaccine design and translational immunotherapy.

## 1 Introduction

Pancreatic cancer is a highly aggressive malignancy, with a five-year relative survival rate long remaining below 13% (1). Late diagnosis, rapid disease progression, and profound therapeutic resistance collectively contribute to its dismal prognosis, making pancreatic cancer one of the most challenging solid tumors to treat. Surgical resection, including pancreaticoduodenectomy or distal pancreatectomy, remains the only potentially curative option, yet its benefit is largely restricted to patients diagnosed at an early, resectable stage (2).

For patients with unresectable or advanced disease, systemic chemotherapy remains the mainstream of treatment (3). Neoadjuvant chemotherapy has been shown to improve clinical outcomes in selected patients with borderline resectable pancreatic cancer (4, 5). The regimen of gemcitabine combined with nab-paclitaxel and the FOLFIRINOX regimen have, to some extent, extended patient survival (6). Modified FOLFIRINOX protocols in the neoadjuvant setting may further improve the progression-free survival (7, 8). However, chemotherapy-associated toxicity, drug resistance, and non-selective damage to the tumor microenvironment make it difficult to achieve lasting therapeutic effects. Therefore, the development of novel treatment strategies that can precisely target tumors and activate the body’s own immune system has become an urgent need in the field of pancreatic cancer research.

Cancer vaccines represent a novel immunotherapeutic modality designed to elicit tumor-specific immune responses through antigen-directed activation of the adaptive immune system (9). However, pancreatic cancer is a prototypical “immunologically cold” tumor, characterized by extensive desmoplasia, a highly immunosuppressive microenvironment, and inefficient antigen presentation. Although peptide-based, dendritic cell-based, and viral vector-based vaccines have demonstrated favorable safety profiles in clinical studies, their therapeutic efficacy has been limited, highlighting the difficulty of overcoming immune exclusion and suppression with single-antigen or poorly coordinated vaccine designs.

Therapeutic and preventive vaccines constitute the two principal branches of cancer vaccines. Preventive vaccines have achieved substantial clinical success, exemplified by the widespread application of HPV vaccines for cervical cancer prevention (10). In contrast, therapeutic cancer vaccines have only recently attracted increasing academic attention, reflecting their considerable but still underexplored clinical potential (11). Based on delivery platforms and antigen composition, therapeutic vaccines are generally classified into peptide, microbial, cellular, and nucleic acid vaccines (12). Peptide-based therapeutic vaccines utilize short antigenic peptides derived from tumor-associated antigens to induce antigen-specific T-cell responses following presentation by dendritic cells via HLA molecules (13). Clinical feasibility has been demonstrated in pancreatic cancer; for instance, an HLA-A24–restricted KIF20A-derived peptide vaccine was associated with improved prognosis in patients with advanced disease (14). Cellular vaccines, most notably dendritic cell–based immunotherapy, involve ex vivo loading of tumor antigens to enhance tumor-specific immune activation (15). Studies in postoperative and advanced pancreatic cancer have provided early clinical evidence supporting the translational potential of dendritic cell vaccines, particularly WT1-pulsed DC vaccines administered in combination with chemotherapy, which may remodel the tumor microenvironment and enable surgical intervention in selected patients (16–18). Microbial vaccines primarily rely on viral vectors to deliver tumor antigens *in vivo*, thereby eliciting antigen-specific immune responses (19).

Nucleic acid vaccines include two main types: DNA vaccines and mRNA vaccines. The core advantage of DNA vaccines lies in their ability to encode full-length tumor antigens, enabling the targeted recognition of multiple antigen epitopes and thereby efficiently inducing immune responses in the body (20). Compared with DNA vaccines, mRNA vaccines offer simpler production processes and lower manufacturing costs (21), making them more suitable for the research and development of personalized tumor vaccines. On one hand, mRNA can be translated into proteins and, through cellular antigen-processing pathways, generate antigen-presenting cells that are taken up and recognized by dendritic cells in the body, which in turn activate downstream signaling pathways and initiate innate immune responses (22). On the other hand, mRNA in the body can be taken up by antigen-presenting cells and translated into proteins. After presentation via the MHC-I and MHC-II pathways, this respectively activates CD8^+^ cytotoxic T cells and CD4^+^ helper T cells (23). Based on the above advantages, mRNA vaccine research has gradually become a focal point for scientists. Given the suitability of these vaccines for personalized preparation, the first critical step in tailoring specific vaccines for different pancreatic cancer patients is the rational selection of tumor antigens (24).

In pancreatic ductal adenocarcinoma (PDAC), multiple KRAS mutant peptides can be presented by HLA molecules, with G12D, G12V, and G12R representing the most prevalent subtypes. These mutations are associated with poor clinical outcomes and are capable of eliciting mutation-specific T cell receptor (TCR) responses, making them attractive targets for mRNA vaccine development. Early clinical evidence supports this concept: a phase I trial demonstrated that the ELI-002 2P vaccine induced robust KRAS-specific T-cell responses and significantly prolonged recurrence-free survival (RFS) (25). In addition, combination therapy with a KRAS G12V/HLA-A*11:01–targeted mRNA vaccine and pembrolizumab achieved partial responses in patients with advanced solid tumors, as assessed by RECIST 1.1 criteria (26). Collectively, these studies highlight the feasibility of KRAS mutants as tumor-specific antigens for mRNA vaccine–based immunotherapy (27). However, the clinical applicability of KRAS-targeted vaccines remains constrained by HLA restriction and mutation heterogeneity, limiting their benefit to a subset of PDAC patients.

Personalized mRNA vaccines targeting patient-specific neoantigens, such as auto-gene cevumeran, have further demonstrated the capacity to induce durable and functional CD8^+^ T-cell responses and to improve RFS in patients with low tumor burden. Nevertheless, vaccines targeting only one or a limited number of antigens are insufficient to address intra-tumoral heterogeneity, thereby facilitating immune escape and treatment resistance (28). Consequently, increasing attention has been directed toward combination and multi-antigen vaccine strategies. Postoperative administration of personalized vaccines may provide immune coverage during periods of high recurrence risk, while combination with immune checkpoint blockade can enhance T-cell infiltration and remodel the tumor immune microenvironment. More broadly, multi-antigen mRNA vaccines offer the potential to elicit polyclonal immune responses, expand antigenic coverage, and concurrently interfere with key oncogenic processes—including proliferation, invasion, metabolic reprogramming, and regulated cell death—thereby achieving a synergistic antitumor effect through coordinated immune killing and functional pathway inhibition.

**Figure 1:**
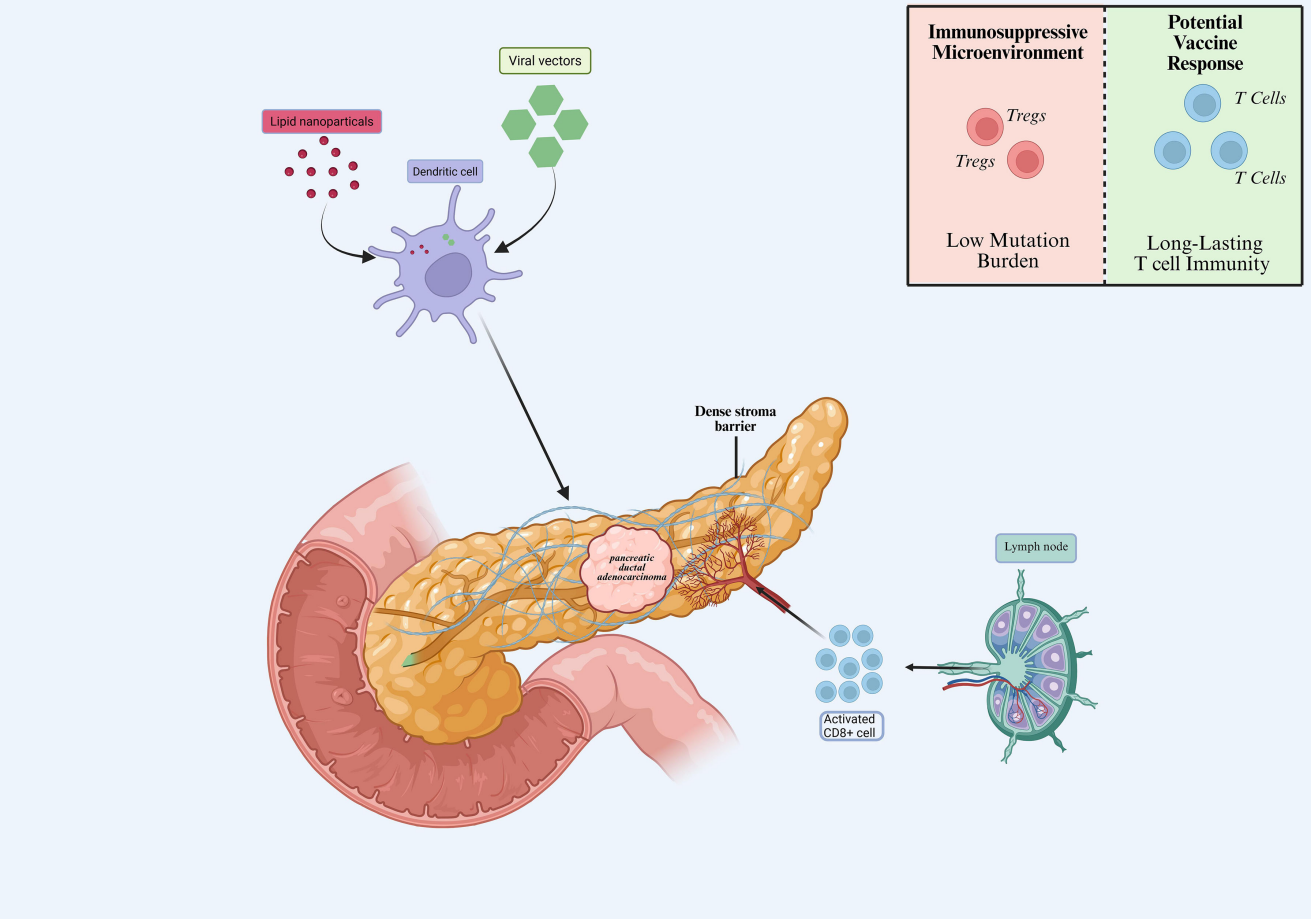
Schematic illustrating the delivery and immune activation mechanism of mRNA vaccines for pancreatic cancer. (created by bioRender). mRNA vaccines are delivered into antigen-presenting cells (e.g., dendritic cells) via lipid nanoparticles or other delivery systems, followed by intracellular translation and antigen processing. The resulting peptides are presented on MHC molecules, leading to activation of CD8^+^ and CD4^+^ T cells and induction of antitumor immune responses. Activated T cells subsequently migrate to tumor sites and mediate tumor cell killing. In pancreatic ductal adenocarcinoma (PDAC), the immunosuppressive microenvironment, including regulatory T cells, low tumor mutation burden, and dense stromal barriers, may limit immune infiltration and vaccine efficacy.

Mechanistically, mRNA vaccines are delivered into antigen-presenting cells, such as dendritic cells, where they are translated into antigenic proteins and processed for presentation via major histocompatibility complex (MHC) molecules. This process leads to the activation of CD8^+^ cytotoxic T cells and CD4^+^ helper T cells, followed by T-cell trafficking to tumor sites and execution of tumor cell killing (29–31). However, in PDAC, the immunosuppressive tumor microenvironment—including regulatory T cells, low tumor mutation burden, and dense stromal barriers—can limit immune infiltration and reduce vaccine efficacy, thereby posing a major challenge to effective antitumor immunity (**Figure 1**) (32, 33).

**Figure 2:**
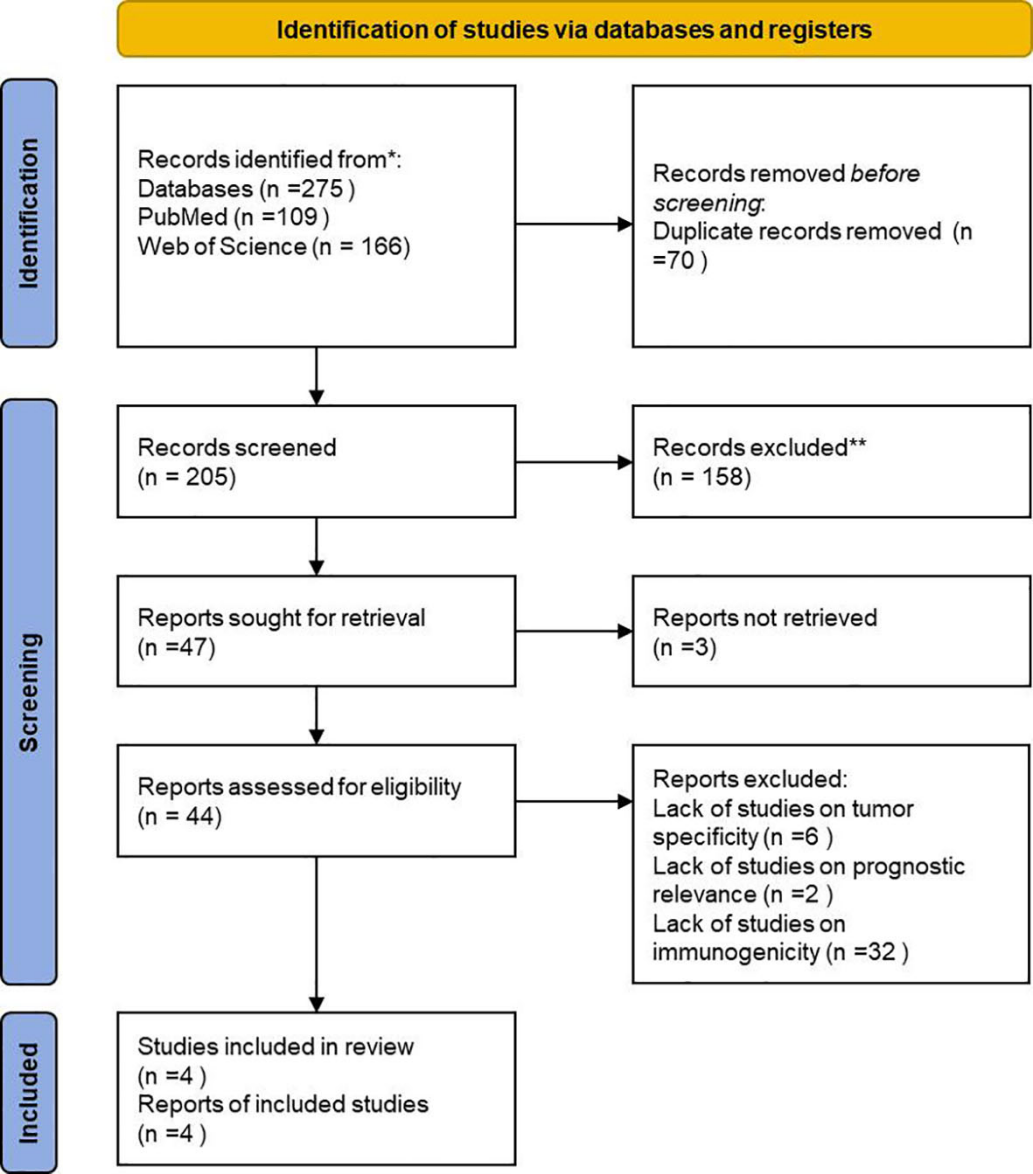
PRISMA flow diagram of literature selection for antigen identification.

This article systematically reviews 16 potential antigens for pancreatic cancer mRNA vaccines (ADAM9, WNT7A, TMOD3, MET, EFNB2, TPX2, AGPS, OSBPL9, KDM5A, NRAS, SCP-1, GAGE, RAB5A, ANO6, CHMP2B, PAK2). Literature searches were performed in PubMed and Web of Science using the keywords “pancreatic cancer” AND “mRNA vaccine” (**Figure 2**). A total of 109 records from PubMed and 166 from Web of Science were initially identified. After removing duplicates and screening titles and abstracts, studies focusing on pancreatic cancer–associated antigens were selected for further evaluation. Full-text assessment was then conducted based on predefined criteria, including tumor specificity, prognostic relevance, and immunological association. Ultimately, four core studies were included for antigen-focused analysis, from which 16 candidate antigens were systematically summarized and further evaluated (**Table 1**). It conducts a comprehensive analysis of their feasibility as vaccine antigens from multiple perspectives, including expression characteristics, molecular functions, immunogenicity, and potential safety risks (34–37).

**Table 1:** Screening and prioritization of 16 candidate antigens for pancreatic cancer mRNA vaccines.

Study	Antigen	Design	Selection	Ref.
Xing Huang et al.	ADAM9、EFNB2、MET、TMOD3、TPX2、WNT7A	Retrospective database review	Tumor tissue-specific overexpression, high-frequency gene mutation, significant correlation with poor prognosis of patients, positive correlation with antigen-presenting cell infiltration	(34)
Ting Yan et al.	AGPS、KDM5A、NRAS、OSBPL9	Retrospective database review	Tumor tissue-specific overexpression, high-frequency gene mutation, significant correlation with poor prognosis and advanced clinical stage of patients, ferroptosis-related gene signature + positive correlation with antigen-presenting cell infiltration	(35)
Boris Kubuschok et al.	SCP-1、GAGE	Prospective Basic Medical Experimental Study	Tumor tissue-specific overexpression, RT-PCR result reproducibility validation, protein-level expression confirmation	(36)
Qiaowei Lin et al.	ANO6、PAK2、CHMP2B、RAB5A	Retrospective database review	Tumor tissue-specific overexpression, high-frequency gene mutation, significant correlation with poor prognosis of patients, pyroptosis-related signature, positive correlation with antigen-presenting cell infiltration	(37)

To better contextualize the rationale for antigen prioritization, current mRNA vaccine strategies in pancreatic cancer can be broadly categorized into three main approaches: personalized neoantigen vaccines, mutation-targeted vaccines (e.g., KRAS), and tumor-associated antigen–based vaccines. Personalized neoantigen vaccines have demonstrated the ability to induce durable, patient-specific T-cell responses, particularly in early-stage or minimal residual disease settings, and have shown promising clinical benefit in pancreatic cancer (38). KRAS mutation–targeted vaccines provide high tumor specificity but are limited by HLA restriction and mutation heterogeneity, although early studies have demonstrated mutation-specific T-cell responses and preliminary clinical activity (39). In contrast, tumor-associated antigens offer broader applicability but often face challenges related to insufficient immunogenicity and immune tolerance (29). A structured comparison of these representative strategies, including their delivery platforms, immunological responses, and clinical outcomes, is summarized in **Table 2**. These limitations highlight the need for a more comprehensive antigen selection framework that integrates tumor specificity, functional relevance, and immunogenic potential. Notably, while tumor-associated antigens may exhibit relatively lower intrinsic immunogenicity compared to neoantigens, their stable expression and broader patient applicability make them particularly suitable as core components in multi-antigen vaccine strategies (29).

**Table 2:** Representative mRNA vaccine strategies in pancreatic cancer.

Antigen type	Representative targets	Platform	Immunological outcomes	Clinical outcomes	Limitations	Ref
Personalized neoantigens	Patient-specific neoantigens (e.g., autogene cevumeran)	mRNA-LNP	Generate a large number of safe, feasible and durable neoantigen-specific T cells	Improved recurrence-free survival (RFS) in resected PDAC	Limited clinical validation	(38)
KRAS mutations	KRAS G12D, G12V	mRNA-LNP/peptide-based	Strong CD8^+^ T-cell responses and memory formation	activation Partial clinical responses reported	Individualized production, time-consuming	(39)
Tumor-associated antigens	ADAM9, MET, TPX2	mRNA-LNP	Variable immunogenicity, often limited T-cell activation	Limited clinical validation	Immune tolerance, lower specificity	(29)

Building upon these considerations, antigen prioritization was further guided by a multi-parameter evaluation framework integrating tumor specificity, functional relevance, immune association, and available immunogenic or epitope-level evidence. By comparing the strength of evidence and translational potential of different antigens, this review aims to provide a theoretical basis for the optimization of antigens and the design of multi-antigen combinations for pancreatic cancer mRNA vaccines, and to guide future experimental research and clinical translation.

## 2 Overview of screening strategies for pancreatic cancer mRNA vaccine antigens

The development of mRNA vaccines for pancreatic cancer relies on the rational selection of antigens. In recent years, antigen selection strategies have shifted from simple expression difference analysis to comprehensive multi-dimensional evaluation. Most studies initially rely on public transcriptomic databases, such as TCGA and GTEx, to identify genes that are significantly overexpressed in pancreatic cancer compared with normal tissues and are associated with unfavorable clinical outcomes. This expression-based approach ensures tumor relevance at a population level but provides limited insight into the immunological suitability of candidate antigens. To address this limitation, subsequent studies have incorporated features of the tumor immune microenvironment, prioritizing antigens that correlate positively with the infiltration of antigen-presenting cells, including dendritic cells, B cells, and macrophages. Such immune-informed strategies increase the likelihood that selected antigens can be effectively processed and presented, thereby enhancing vaccine-induced immune activation. More recently, pathway-oriented screening frameworks have been introduced, integrating tumor-associated regulatory processes such as ferroptosis and pyroptosis into antigen selection. While this strategy broadens the spectrum of candidate antigens and links vaccination to functionally relevant tumor vulnerabilities, it also introduces substantial heterogeneity in the biological maturity and translational feasibility of different targets. Consequently, systematic stratification and comparative evaluation are required to distinguish clinically actionable vaccine antigens from exploratory candidates.

**Figure 3:**
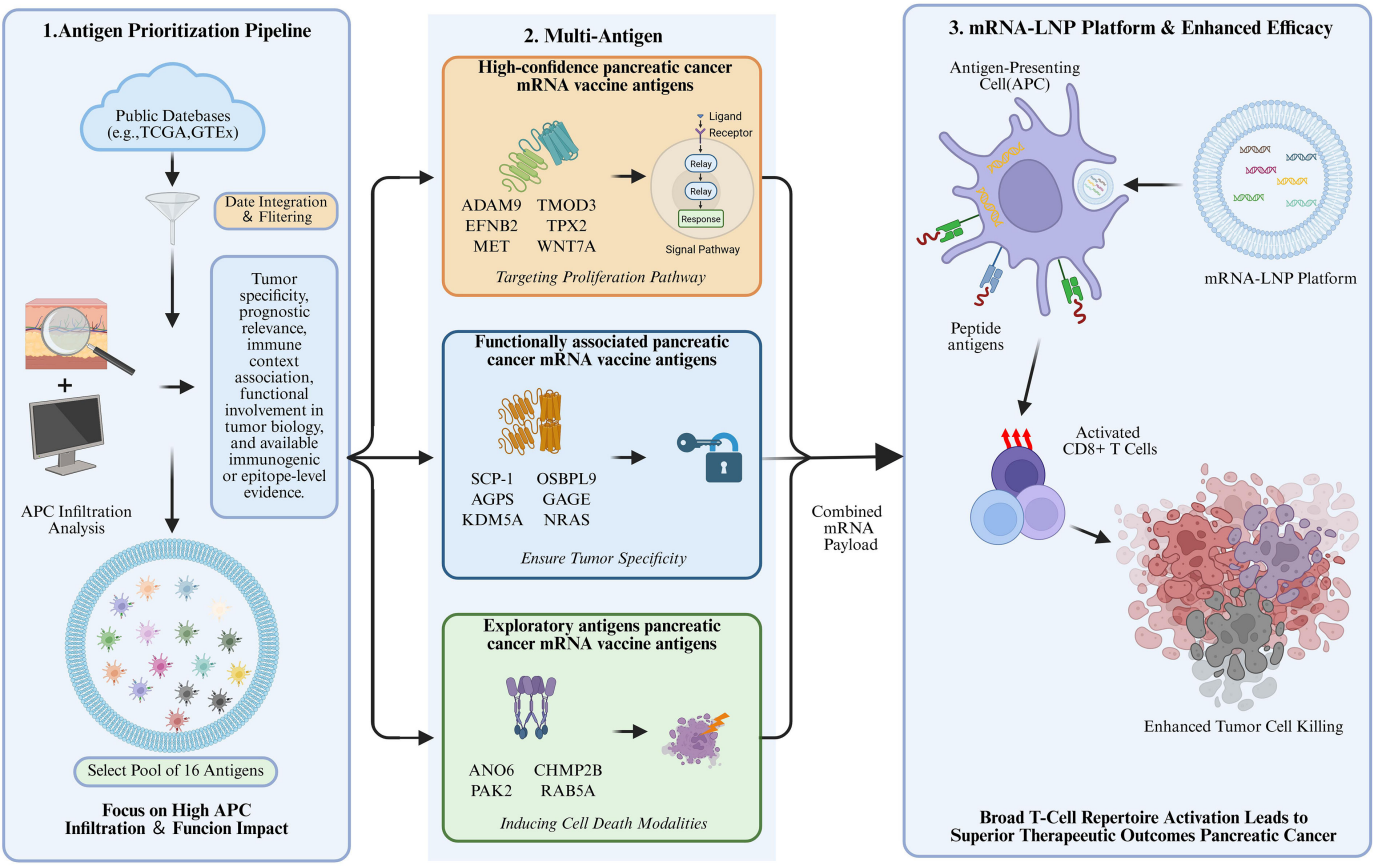
Schematic overview of the antigen prioritization and multi-antigen mRNA vaccine design framework for pancreatic cancer. (created by bioRender) This figure illustrates a three-step strategy for identifying and applying candidate antigens for pancreatic cancer mRNA vaccines. (1) Antigen prioritization: Candidate antigens are screened from public databases (e.g., TCGA, GTEx) based on tumor-specific expression, prognostic relevance, immune context association, functional involvement, and available immunogenic or epitope-level evidence, with emphasis on antigens linked to antigen-presenting cell (APC) infiltration. (2) Antigen stratification: Selected antigens are categorized into high-confidence, functionally associated, and exploratory groups according to evidentiary strength and biological roles. (3) Vaccine design and immune activation: Multiple antigens are combined into an mRNA payload and delivered via lipid nanoparticles (LNPs) to antigen-presenting cells, leading to antigen presentation, CD8^+^ T-cell activation, and enhanced tumor cell killing, ultimately promoting broader T-cell responses and improved therapeutic outcomes.

Most studies use public databases such as TCGA and GTEx as a foundation, comparing transcriptomic differences between tumor and normal tissues to identify candidate genes that are significantly upregulated in pancreatic cancer and associated with poor prognosis. This strategy ensures the tumor relevance of antigens on a macro level but does not directly reflect their immunological value. To further organize these heterogeneous screening strategies, we constructed an integrated prioritization framework for pancreatic cancer mRNA vaccine antigens, as shown in **Figure 3**. This figure summarizes the overall logic of our review, including antigen selection, evidence-based stratification, and the conceptual basis for multi-antigen mRNA vaccine design.

## 3 High-confidence pancreatic cancer mRNA vaccine antigens

Based on current evidence, a subset of pancreatic cancer antigens exhibits a relatively complete chain of support encompassing tumor-specific expression, functional involvement in malignant progression, and immunogenic potential. These antigens therefore represent high-confidence targets with the greatest translational relevance for pancreatic cancer mRNA vaccine development.

### 3.1 MET

MET, as a classic receptor tyrosine kinase (RTK) (40) and the specific receptor for hepatocyte growth factor (HGF), has been extensively studied and is confirmed to be closely associated with the abnormal biological behavior of malignant tumors (41). After binding with HGF, MET undergoes phosphorylation, which in turn activates multiple downstream signaling pathways. This not only mediates the activation of signal transducers and activators of transcription (STATs) (42) but also triggers cascade reactions in the ERK and PI3K/AKT signaling pathways (43, 44). The HGF/c-MET signaling pathway is not only widely involved in the occurrence and development of pancreatic cancer (45, 46), but also plays a regulatory role in physiological processes such as embryonic development and wound regeneration (47). At present, research on the HGF/c-MET signaling pathway is relatively systematic and in-depth, and its mechanisms in pancreatic cancer progression are also well elucidated. From the perspective of vaccine antigen selection, it is also important to consider the physiological functions this pathway plays in normal and benign disease processes. In a mouse model study, researchers constructed MET gene knockout mice and induced pancreatitis via alcohol intervention. The results showed that knockout of the MET gene reduced the threshold for irreversible damage to pancreatic tissue, suggesting that the HGF/c-MET signaling pathway plays a regulatory role in the repair process during inflammatory responses (48). In addition, the HGF/c-MET pathway may also have certain regulatory effects during pancreatic embryonic development and functional maturation (49, 50). Beyond its role in acute and chronic inflammatory processes such as pancreatitis, c-Met also holds considerable potential as a therapeutic target in pancreatic cancer. The malignant proliferation and self-renewal of tumor tissues depend on the maintenance of cancer stem cell (CSC) stemness. By comparing the number of CSCs and tumor growth rates in mouse models with different levels of c-Met expression, it was confirmed that c-Met is an essential regulatory molecule in the growth and metastasis of mouse pancreatic tumors, thereby clarifying its potential as a therapeutic target in pancreatic cancer (51). Application of c-Met inhibitors can significantly enhance the therapeutic effect of vascular endothelial growth factor receptor (VEGFR) blockers. This effect can further intensify the hypoxic microenvironment within tumor tissues, promote apoptosis of tumor cells, and ultimately effectively inhibit tumor invasion and metastasis (52). In addition to demonstrating clear efficacy in patients with PDAC, the most common subtype of pancreatic cancer, the combination of c-Met inhibitors and VEGFR inhibitors can also effectively suppress invasion and metastasis in pancreatic neuroendocrine tumors (53). From a vaccine perspective, MET is attractive due to its marked overexpression in pancreatic cancer, with c-MET and HGF mRNA levels significantly exceeding those in normal pancreatic tissue (54). The immunogenicity of this molecule has been clearly demonstrated, making it a highly promising candidate antigen for pancreatic cancer vaccine development. However, since the signaling pathways, it participates in are also involved in the maintenance of homeostasis and damage repair in normal tissues, the specific immune targeting effect induced by the vaccine may cause non-specific attacks on normal tissues, posing potential safety risks. In contrast, its combination with other potential antigens that are highly expressed in pancreatic cancer can leverage the synergistic targeting effect of multiple antigens to greatly enhance the recognition specificity and targeting accuracy of the vaccine for pancreatic cancer tissues. Meanwhile, it can activate the body’s anti-tumor immune response through multiple targets to strengthen the overall immune killing effect. Furthermore, this combination strategy can effectively reduce the probability of immune escape induced by a single antigen and mitigate the safety risks associated with single-targeting, thus enabling the vaccine to further improve the safety and reliability of its clinical application while maintaining robust immune activity.

### 3.2 SCP-1

Synaptonemal Complex Protein 1 (SCP-1), also known as HOM-TES-14, is a protein with a unique tissue expression profile and functional characteristics and belongs to the cancer-testis antigen (CT antigen) family. The expression of SCP-1 is significantly associated with liver metastasis of colorectal cancer, and its expression levels are closely linked to malignant phenotypes such as vascular cancer emboli formation, tumor invasion depth, and lymph node metastasis (55). SCP-1 may play a common regulatory role in the metastatic processes of various tumors (56, 57), thereby providing important insights for investigating the function of SCP-1 in prognosis assessment and the metastatic mechanism of pancreatic cancer. Aberrant expression of SCP-1 in tumor tissues may be involved in regulating the process of meiosis in tumor cells and play a role in the orderly progression of the tumor cell cycle (58). SCP-1 is highly expressed in pancreatic cancer tissues but is not detected in normal pancreatic tissues; however, its expression can also be detected in specimens of chronic pancreatitis, which may support the notion that chronic pancreatitis is a precancerous lesion of pancreatic cancer (36). Although among 130 pancreatic cancer samples, cases of high SCP-1 expression accounted for only 19% (59), the protein’s specific expression characteristics in tumor tissues still suggest its potential as an antigenic target for pancreatic cancer vaccine development. SCP-1 possesses the attributes of a high-quality tumor antigen, meeting the requirements for antigens to “target tumors while sparing normal tissues.” On the other hand, its functional T cell epitope p635-649 can induce specific CD4^+^ T cell responses, thereby activating cytotoxic T lymphocytes (CTLs), macrophages, and other immune effector cells to generate a robust antitumor cellular immune response. Additionally, protein fragments covering p635-649 can be naturally processed and presented by both autologous and allogeneic dendritic cells, and this epitope exhibits high binding affinity to MHC-II molecules, ensuring that SCP-1 epitopes can efficiently activate immune cells *in vivo (*60). These features enable the activation of cytotoxic T lymphocytes and other immune effector cells, supporting SCP-1 as a high-quality antigen for vaccine-based immunotherapy.

### 3.3 GAGE

Members of the GAGE family represent classical CT antigens originally identified in melanoma and recognized by cytotoxic T lymphocytes (61). GAGE expression has been reported across various malignant tumor tissues, including melanoma (62), neuroblastoma (63), and hepatocellular carcinoma (64). GAGE, like SCP-1, belongs to the CT antigen family. Interestingly, both are highly expressed in pancreatic cancer tissues and chronic pancreatitis tissues, but absent in normal tissues, suggesting that GAGE may share similar biological characteristics with SCP-1 (65). Notably, high expression of GAGE may not be significantly associated with invasion or metastasis in breast cancer (66), and its role and regulatory mechanisms in other tumor types still require further validation. At present, the specific molecular mechanisms by which GAGE proteins exert carcinogenic effects remain to be elucidated. Some researchers have proposed that GAGE proteins may be recruited to the nuclear membrane, mediating their binding to intracellular DNA and thereby regulating the expression state of chromatin. In germ cells, this protein can mediate the transition of cells from a somatic state to a germ cell state (67, 68), a feature that suggests GAGE may also play a role in tumorigenesis through DNA binding. However, this hypothesis still requires further experimental validation. Currently, research on the oncogenic molecular mechanisms of GAGE is still limited, but existing studies have shown that elevated expression may affect the sensitivity of tumor cells to radiotherapy and chemotherapy, thus participating in the regulation of tumor drug resistance (68, 69). Based on this, developing tumor vaccines targeting GAGE for patients resistant to radiotherapy and chemotherapy may become a new research direction in this field. A study on cancer vaccines for renal cell carcinoma proposed the feasibility of using GAGE as a tumor vaccine antigen target (70). This research demonstrated the antigenic potential of GAGE from multiple perspectives: drug-induced expression of GAGE in tumor cells, the capability of induced GAGE proteins to be recognized by specific T cells to mediate anti-tumor immune responses, conversion of “cold tumors” to “hot tumors” through drug-induced expression of GAGE and other CT antigens, and its expression restricted to tumor tissues but not in normal tissues, thereby effectively avoiding off-target immune toxicity.

### 3.4 PAK2

p21-activated kinase 2 (PAK2) is a serine/threonine kinase involved in cytoskeletal remodeling, cell migration, and programmed cell death (71, 72). In malignant tumors, its expression is upregulated in malignant tumors such as gastric cancer (73), lung adenocarcinoma (74), and pancreatic cancer (75). As a highly expressed protein in pancreatic cancer tissue, studies using bioinformatics have found that PAK2 has the potential to promote angiogenesis in cancer cells and can drive the epithelial-mesenchymal transition (EMT) process by activating the TGF-β signaling pathway. Meanwhile, PAK2 decreases the differentiation level of cancer cells, further enhancing their malignancy (76, 77), indicating its crucial regulatory role in the proliferation and metastasis of pancreatic cancer. Further research has confirmed that in the malignant progression of PDAC, PAK2 serves as a core downstream effector mediating cancer cell migration and metastasis; PKM2, HSP90, and PAK2 can assemble into a functional complex, which, through synergistic action, regulates the metastatic process of PDAC (78). Pyroptosis is a form of programmed cell death dependent on inflammasomes and caspases, and its function in tumorigenesis and development has been increasingly revealed in recent years. Studies have shown that overexpression of the long non-coding RNA FAF can significantly alleviate cardiomyocyte pyroptosis by upregulating PAK2 expression, suggesting that PAK2 serves as a downstream effector with negative regulatory function in the pyroptosis process (79). Accordingly, it is speculated that in pancreatic cancer tissue, upregulation of PAK2 expression may also inhibit tumor cell pyroptosis, thereby participating in the regulation of pancreatic cancer malignancy, although this requires further research for confirmation. At the immunogenic level, PAK2 can induce a strong CD8^+^ T cell immune response in *in vitro* models (80). As a positive regulatory factor in TCR signaling-dependent T cell activation, PAK2, through its kinase activity and unique structural domains mediating molecular interactions, regulates the signaling and functional molecule expression associated with T cell activation (81). Its upregulation can promote the increased expression of immune-related molecules, suggesting that PAK2 may activate specific immune responses against tumor cells. PAK2 exhibits significant immunogenic advantages, can effectively activate CD8^+^ T cell immune responses, and positively regulate T cell activation, providing a key guarantee for vaccine immunogenicity. In the future, it is necessary to further clarify the regulatory mechanism of PAK2 in pyroptosis in pancreatic cancer, screen for precise antigen epitopes, and verify the immunogenicity and antitumor activity of PAK2 vaccines through *in vivo* and *in vitro* experiments.

### 3.5 TPX2

TPX2 is a microtubule-associated protein that regulates mitotic spindle assembly and Aurora-A kinase localization, thereby controlling cell-cycle progression (82). Studies have shown that the phosphorylation of TPX2 allows for precise regulation of DNA double-strand break (DSB) repair pathways. Phosphorylated TPX2 can activate the homologous recombination (HR) repair pathway, which performs high-fidelity repair primarily during the S/G2 phases of the cell cycle, while unphosphorylated TPX2 mediates the non-homologous end joining (NHEJ) pathway, participating in the regulation of cell cycle progression (83). This suggests that TPX2 may regulate the malignant proliferation of tumor cells. In addition to potentially participating in tumor proliferation through cell cycle regulation, TPX2 is also speculated to influence angiogenesis in pancreatic cancer, although this effect may be mediated by insulin-like growth factor binding protein 3 (IGFBP-3) (84). Although the specific mechanisms by which TPX2 regulates tumor proliferation and invasion have not yet been fully elucidated, the consensus in the academic community is that TPX2 is abnormally overexpressed in pancreatic cancer tissues. Some researchers have conducted specialized studies on the expression characteristics of TPX2 in pancreatic cancer tissues, and the results indicate that Aurora-A kinase and TPX2 show promise as potential biomarkers for pancreatic ductal adenocarcinoma carrying KRAS mutations (85). Other studies have confirmed that TPX2 can serve as an independent prognostic factor for pancreatic cancer. Through RNA sequencing and survival analysis of public databases, these studies found that TPX2 is significantly overexpressed in pancreatic cancer tissues, and its high expression is closely associated with poor patient prognosis (86, 87). Notably, TPX2 is not expressed in normal pancreatic tissue or pancreatic intraepithelial neoplasia (PanIN), further supporting its value as a specific tumor marker for pancreatic cancer (84). TPX2 has the fundamental characteristics required to serve as a safe and effective vaccine antigen, but the specific molecular mechanisms by which it regulates tumor progression have yet to be clarified, which presents challenges for the precise design and efficacy prediction of vaccines. Stimulation of the TME with antigens and other immunosuppressive factors may trigger CD8^+^ T cell exhaustion, a critical issue that severely restricts the screening of vaccine antigens. In contrast, TPX2 has been demonstrated to play an essential role in maintaining the anti-tumor activity of CD8^+^ T cells (88), and its inherent immunogenicity renders it a promising candidate for combined vaccine development with highly expressed antigens in pancreatic cancer, or for synergistic application with immune checkpoint inhibitors, thereby effectively overcoming the limitations associated with single-antigen vaccines.

## 4 Functionally associated potential vaccine antigens

Beyond high-confidence targets, several candidate antigens exhibit well-defined or putative functional involvement in key biological processes of pancreatic cancer but currently lack robust evidence supporting their immunogenicity. These antigens may represent intermediate-priority candidates with translational potential contingent upon further validation.

### 4.1 ADAM9

ADAM9, a member of the a disintegrin and metalloproteinase family, is a cell-surface endopeptidase aberrantly overexpressed in multiple malignancies, including pancreatic ductal adenocarcinoma (PDAC) (89, 90). Importantly, its expression displays marked histological specificity within pancreatic tumors. A clinical study demonstrated that ADAM9 expression is specific to certain pathological types of pancreatic cancer: among 59 cases of PDAC, the high expression rate reached 98.3% (58/59); among 24 cases of pancreatic acinar cell carcinoma, the high expression rate was only 8.3% (2/24); and all 11 cases of pancreatic neuroendocrine tumors showed no high expression of ADAM9 (91). This differential expression highlights the value of ADAM9 as a PDAC antigen target. Inhibiting ADAM9 is thought to promote KRAS degradation. When ADAM9 is absent, the interaction between KRAS and plasminogen activator inhibitor 1 (PAI-1) is significantly enhanced. PAI-1 acts as a selective autophagy receptor, working synergistically with light chain 3 (LC3), which ultimately mediates the lysosomal degradation of KRAS (92). Another study pointed out that circular RNA ADAM9 (circ-ADAM9) can directly bind to microRNA-217 (miR-217) via sequence complementarity, diminishing its inhibitory effect on protease serine 3 (PRSS3), thereby upregulating PRSS3 expression and ultimately activating the ERK/VEGF signaling pathway (93). The regulatory interactions between ADAM9 and multiple microRNAs are further summarized in **Table 3**. In addition to miR-217, interactions between ADAM9 and microRNAs such as miR-126 (94), miR-520f (95), and miR-489 (96) also play key regulatory roles in suppressing pancreatic cancer cells. Studies based on two PDAC cell lines have confirmed that ADAM9 plays a critical role in mediating cell migration and adhesion to extracellular matrix substrates. At the same time, ADAM9 can significantly promote angiogenesis and has a strong pro-angiogenic effect (97). The high expression of ADAM9 in PDAC can markedly enhance the tumor’s invasive and infiltrative abilities. The expression of ADAM9 has been demonstrated to be associated with T cell dysfunction and exhaustion (98), which indicates that its immunogenicity is insufficient to support its use as a candidate target for pancreatic cancer single-antigen vaccines. However, this molecule is characterized by specific high expression in PDAC, enabling it to serve as an effective target for the precise tumor tissue targeting of combination vaccines; the core potential of ADAM9 as a candidate molecule for tumor vaccines stems precisely from this property of relatively specific high expression.

**Table 3:** Regulatory functions of microRNAs.

microRNA	ADAM9 expression correlation	Regulated signaling pathway	Upstream/downstream regulator	Ref.
miR-217	Negative correlation	circ-ADAM9/miR-217/PRSS3 →ERK/VEGF	Downstream	(93)
miR-126	Negative correlation	miR-126→ADAM9 3′-UTR→EMT	Upstream	(94, 99)
miR-489	Negative correlation	KRAS→ NF-κB → YY1→ MIR489→ miR-489→ ADAM9/MMP7	Upstream	(96)
miR-520f	Negative correlation	miR-520f→ ADAM9/TGFBR2→ CDH1、SNAI2/ZEB1/ZEB2→ EMT	Upstream	(95)

### 4.2 EFNB2

The erythropoietin-producing hepatocyte receptor (Eph receptor) is known as the largest family of receptor tyrosine kinases (RTKs) (100). Ephrin-B2 (EFNB2), as an important member of this family, can specifically bind to the EphB4 receptor. The EphrinB2/EphB4 signaling axis they form is one of the core pathways regulating angiogenesis and plays a key role in the proliferation of microvascular endothelial cells, the maturation of new blood vessels, and functional remodeling processes (101). EFNB2 has also been found to be abnormally overexpressed in a variety of malignant tumor tissues. The expression level of EFNB2 in hepatocellular carcinoma (HCC) tissues is significantly higher than in normal liver tissue. Similarly, in intrahepatic cholangiocarcinoma (CCA) and colorectal cancer liver metastases (CRLM), the expression of EFNB2 is also markedly upregulated compared to normal liver tissues (102). In laryngeal squamous cell carcinoma, upregulation of integrin α5 (ITGA5) can positively regulate the expression level of EFNB2, thereby participating in the tumorigenesis process (103). In gastric cancer, EFNB2 can promote tumor cell proliferation by activating the Wnt/β-catenin signaling pathway (104). EFNB2 has been confirmed to have the potential to serve as a therapeutic target in pancreatic cancer, as it plays a key regulatory role in the occurrence and development of tumors (105). In pancreatic ductal adenocarcinoma tissues, EFNB2 expression is significantly upregulated, and its expression level is strongly correlated with the clinical stage of PDAC and Ki67 proliferation index. Functional experiments have confirmed that downregulation of EFNB2 expression can mediate G0/G1 cell cycle arrest by activating the p53/p21 signaling pathway, thereby inhibiting tumor cell proliferation. At the same time, knocking down EFNB2 can block the EMT process, resulting in a significant reduction in the invasive capacity of PDAC cells (106). In studies regarding radiotherapy resistance in pancreatic cancer, it was found that inhibiting the activity of a disintegrin and metalloproteinase 10 (ADAM10), combined with gene knockout of EFNB2 in fibroblasts of the tumor microenvironment, can effectively reduce the metastatic capability of pancreatic cancer after radiotherapy (107). The expression of EFNB2 is closely associated with the enhanced cytotoxic activity of CD8^+^ T cells and elevated immune cell infiltration in the TME, and it can effectively promote T cell proliferation (108). This molecule is expressed on the surface of T cells and, upon cross-linking with the T cell receptor (TCR), participates in T cell costimulation (109). However, research on the immunogenicity of EFNB2 remains scarce to date, with particularly limited relevant investigations in the field of pancreatic cancer, and its potential application as a candidate target for pancreatic cancer vaccines thus requires further verification through subsequent related experiments.

### 4.3 KDM5A

KDM5A (JARID1A/RBP2) is a histone demethylase that is significantly overexpressed in various malignant tumors, including non-small cell lung cancer (110), breast cancer (111), and prostate cancer (112). In pancreatic cancer tissues, KDM5A is also markedly overexpressed, and its expression level is positively correlated with tumor clinical staging, patient prognosis, and other indicators. In pancreatic cancer tissues with high KDM5A expression, the infiltration of B cells, CD8^+^ T cells, and other immune lymphocytes is significantly increased (113, 114). Based on these findings, some researchers have further explored and discovered that KDM5A may be involved in the hypoxia-mediated metastatic pathway of pancreatic cancer. Hypoxic environments can induce upregulation of NADPH oxidase 4 (NOX4), leading to inactivation of KDM5A, which in turn increases histone methylation levels. Ultimately, this regulates the expression of the EMT-related gene SNAIL1, promoting the invasion and metastasis of pancreatic cancer (115). Additional studies in neuroendocrine and other tumor types suggest that KDM5A modulates histone methylation states and cooperates with tumor suppressors such as MEN1 to regulate tumor growth (116). A study related to endometrial cancer elucidated the specific mechanism by which KDM5A regulates ferroptosis. Downregulation of KDM5A expression promotes the upregulation of NSUN2, which in turn enhances the m5C modification of SLC7A11 mRNA. The enhanced m5C modification increases SLC7A11 expression, ultimately inhibiting lipid peroxidation within the cell and thereby blocking ferroptosis (117). Another study focusing on “persister cells” resistant to chemotherapy in head and neck cancer indicated that increased KDM5A expression can mediate downregulation of MPC1, promoting the formation of mesenchymal phenotypes in cancer cells and resulting in antioxidant program defects, ultimately facilitating the occurrence of ferroptosis (118). Previous studies have confirmed that upregulating the expression level of KDM5A in combination therapy can significantly enhance the overall recruitment efficiency of activated CD8^+^ T cells to tumor tissues (119). Another study demonstrated that NSUN6 deficiency in PDAC induces immunosuppression through the m5C-KDM5A-CCL2 signaling axis, thereby impairing the anti-tumor immune response mediated by CD8^+^ T cells in PDAC (120). Furthermore, additional research has revealed a negative correlation between the expression level of KDM5A and that of genes associated with the antigen processing and presentation pathway (HLA-A, HLA-B) (121). Although KDM5A is widely involved in the oncogenesis and progression of pancreatic cancer, its intrinsic immunogenicity as a single antigen remains to be further verified.

### 4.4 ANO6

ANO6, also known as transmembrane protein 16F (TMEM16F), is a member of the calcium-activated chloride channel and lipid remodelase family. It possesses ion flow regulation functions and has been confirmed as a critical molecule indispensable to the calcium-mediated membrane phospholipid scrambling process (122). In addition, abnormal expression of this protein in various malignancies has been verified through research: its expression is downregulated in prostate cancer (123), ovarian cancer (124), and neuroblastoma (125), while upregulated in pancreatic cancer (126) and gastric adenocarcinoma (127). Regarding the mechanism by which ANO6 regulates pancreatic cancer progression, some studies suggest that it may mediate the transport of lipids (especially cholesterol) taken up by fibroblasts to tumor cells, supplementing their essential lipid requirements. At the same time, it can promote fibroblasts to transfer excess cholesterol to cytotoxic T cells, thereby inhibiting their antitumor immune activity (128). Furthermore, other studies have shown that upregulation of ANO6 expression can activate the downstream ERK signaling pathway, promoting tumor proliferation and invasion (129). In terms of immune response, ANO6 can mediate phosphatidylserine disorder to interfere with macrophage polarization (130). ANO6 not only participates in channel transport functions but can also regulate cell death processes. It can be involved in the formation of membrane pores, thereby triggering a series of pathological morphological changes such as massive membrane erosion, cell swelling, and membrane disintegration (131). Other research indicates that ANO6 plays a synergistic role in reactive oxygen species (ROS)-mediated cell death, and this effect is mediated through the ferroptosis pathway (132). Although these functional characteristics render ANO6 a highly functionally relevant candidate antigen for tumor vaccine development, its immunogenic features directly associated with vaccine development have not yet been investigated systematically and in depth. During the host anti-infectious immune response, ANO6 can negatively regulate T cell proliferation by mediating TCR degradation and immune signal termination (133); in contrast, other studies have confirmed that this molecule can also mediate bystander TCR-CD3 membrane dissociation at the immunological synapse, thereby positively enhancing the activation of T cells (134). At present, the immunoregulatory role of ANO6 in the tumor microenvironment remains largely unknown, and there is a particular lack of *in vitro* and *in vivo* experimental data and mechanistic studies that directly verify its tumor-associated immunogenicity. Whether it can serve as a candidate target for tumor vaccines thus requires further exploration.

### 4.5 RAB5A

RAB5A is a small GTPase that regulates early endosome formation and intracellular trafficking, processes essential for tumor cell migration, invasion, and metastasis (135–140). In pancreatic cancer, RAB5A is consistently overexpressed and associated with poor prognosis, enhanced lymphatic metastasis, and activation of Wnt/β-catenin signaling (141). There is evidence that this molecule has an association with WNT7A, and the two may work together to regulate this signaling pathway. Therefore, tumor vaccine strategies targeting the combined antigenic sites of RAB5A and WNT7A, or even multiple antigenic sites, offer a new research direction for immunotherapy in pancreatic cancer. Furthermore, in pancreatic cancer with KRAS gene mutations, the expression level of RAB5A is significantly upregulated, which, by regulating the formation and fusion of endosomes, leads to abnormal early endosome enlargement and thus participates in the malignant progression of pancreatic cancer (142). In addition, upregulation of RAB5A expression can enhance autophagic activity in cells, possibly promoting cancer cell survival and ultimately contributing to carcinogenesis (143, 144). As a core GTPase that regulates early endosome formation and transport, RAB5A plays a widespread role in the signal transduction and substance transport of various immune cells. Although research has shown that its expression level is positively correlated with various immune cells and stromal cells (37), studies on its specific mechanisms of action are still relatively limited. Although RAB5A is functionally linked to tumor progression and immune–stromal interactions, its ubiquitous role in vesicular trafficking across multiple cell types presents a major challenge for antigen specificity. At present, its suitability as a vaccine antigen remains speculative and requires rigorous immunogenic assessment.

### 4.6 TMOD3

TMOD3 is an actin-regulatory protein whose role in pancreatic cancer has only recently begun to emerge. Initial database-driven studies identified TMOD3 as an overexpressed protein associated with poor survival in pancreatic cancer, but mechanistic insights were limited (145, 146). More recent clinical analyses have confirmed that elevated TMOD3 expression correlates with unfavorable prognosis and enhanced invasive capacity in pancreatic cancer patients (147). In the metastatic process of bladder cancer, TMOD3 can activate the MAPK signaling pathway, induce phosphorylation of ERK1/2 within the pathway, and thereby enhance the invasive capability of tumor cells (148). Similarly, in liver cancer, TMOD3 can promote tumor proliferation by activating related signaling pathways (149). Based on this, it is speculated that TMOD3 may also mediate tumor proliferation and metastasis in pancreatic cancer by activating the MAPK signaling pathway, though this hypothesis has not yet been confirmed by relevant research. TMOD3 can promote the binding of actin and myosin filaments and inhibit the depolymerization of their complexes (150), a biological function that may be closely related to the migration and invasiveness of tumor cells (151). In patients with pancreatic cancer carrying KRAS gene mutations, TMOD3 can promote the polymerization of F-actin and inhibit ferroptosis in tumor cells, thereby inducing resistance to PD-1 antibodies (152). The study also suggested that an FDA-approved drug could be used to target and inhibit the expression of TMOD3, making TMOD3 a potential molecular target for pancreatic cancer targeted therapy. At present, studies on TMOD3 are still relatively scarce, especially in the field of pancreatic cancer; and the few existing studies have not differentiated between different pathological types such as pancreatic ductal adenocarcinoma and pancreatic acinar cell carcinoma.

## 5 Exploratory antigens and their methodological significance

A subset of candidate antigens has been proposed primarily on the basis of bioinformatics-driven screening or indirect functional associations, whereas their immunological relevance in pancreatic cancer remains insufficiently defined. Importantly, these candidates highlight the methodological trade-offs of current prioritization frameworks—expanding the antigen repertoire by integrating immune infiltration and pathway signatures, yet increasing uncertainty in biological validity and translational feasibility.

### 5.1 OSBPL9

OSBPL9, an oxysterol-binding protein–like family member implicated in lipid transport and signaling, has emerged as a candidate mainly through database-based analyses (153). Notably, OSBPL9 is reported to be downregulated in PDAC compared with normal pancreas (154), mirroring observations in hepatocellular carcinoma (155), and has been linked to dysregulated lipid metabolism in pancreatitis (156). In neuroblastoma, OSBPL9 is potentially implicated in the regulation of the tumor microenvironment, yet research into the specific functional relationship between OSBPL9 and the tumor microenvironment remains scarce to date (157). A study constructed a prognostic prediction model for MYCN-amplified neuroblastoma based on the ferroptosis mechanism; through the analysis of the TARGET database, four ferroptosis-related genes were screened and identified, among which OSBPL9 was confirmed to be significantly correlated with patient prognosis (158) and in a ferroptosis-oriented PDAC antigen-screening framework, OSBPL9 was prioritized owing to its association with antigen-presenting cell infiltration (35). Although OSBPL9 has been proposed to participate in sterol–phospholipid metabolic integration and ferroptosis regulation (159), the predominance of in silico evidence, unclear mechanism, and its atypical “low-in-tumor” expression pattern currently limit its candidacy as a reliable vaccine antigen. Nevertheless, OSBPL9 exemplifies how pathway-oriented screening can surface unconventional targets that warrant targeted mechanistic and immunogenic validation, and it has been explored in antigen discovery efforts beyond PDAC (160).

### 5.2 NRAS

Compared with KRAS, NRAS has received relatively limited attention in pancreatic cancer. NRAS mutations appear exceedingly rare in pancreatic cancer cohorts, reinforcing KRAS as the dominant mutational driver (161). Interestingly, limited clinical data suggest that NRAS expression level—rather than mutation status—may correlate with prognosis in PDAC, raising the possibility of context-dependent, non-canonical roles (162). NRAS can engage canonical RAF–MEK–ERK and PI3K–AKT–mTOR signaling, and emerging studies in other cancers link NRAS biology to ferroptosis regulation and therapy response (163–165). NRAS has also been identified within immune-related gene sets, including NK-cell–associated signatures in other tumor types (166). However, direct evidence connecting NRAS expression to antigen presentation, epitope immunogenicity, or vaccine responsiveness in PDAC is currently lacking, positioning NRAS as an exploratory candidate that primarily underscores the limitations of extrapolating from pan-cancer signaling homology.

### 5.3 CHMP2B

CHMP2B is a component of the ESCRT-III machinery involved in endosomal trafficking, membrane remodeling, and autophagy (167). In oncology, CHMP2B expression has shown heterogeneous patterns across tumor types and has been incorporated into immune- and autophagy-related prognostic models, including those for pancreatic cancer (168–170). These observations imply a potential link to tumor–immune interactions and autophagic homeostasis, processes relevant to antigen processing and immune regulation. Nonetheless, CHMP2B remains largely unsupported by experimental evidence in the context of vaccine antigen development, and its biological role in PDAC immunity is insufficiently characterized, making it a low-priority antigen until functional and immunogenic studies become available.

### 5.4 WNT7A

The WNT protein family belongs to a highly conserved family of secreted glycoproteins, among which the Wnt/β-catenin signaling pathway is a classic signaling route that mediates the regulation of various biological processes such as cell apoptosis, invasion, and proliferation (171). WNT7A can regulate the Wnt/β-catenin signaling pathway in endometrial cancer (172), head and neck squamous cell carcinoma (173), bladder cancer (174), and pancreatic cancer (175), thereby mediating the growth and metastasis of tumor cells. High expression of WNT7A may enhance the migratory ability of pancreatic ductal adenocarcinoma (PDAC) cells, and this effect may be achieved by inducing the epithelial-mesenchymal transition (EMT) process in PDAC cells (175). WNT7A can serve as a key component of the miR-4723/WNT7A signaling axis, with CAV2 able to regulate the activity of this axis by inducing the EMT process, thereby promoting tumor cell proliferation (176). A 2025 clinical study involving 96 PDAC patients showed that 50% of patients exhibited abnormally high expression of circPHF14. As a downstream target of circPHF14, WNT7A can bind to circPHF14 through a specific nucleic acid sequence in its mRNA, thus maintaining the stability of WNT7A mRNA, ultimately activating the Wnt signaling pathway and promoting the progression of PDAC cells (177). WNT7A is abnormally expressed in PDAC, whereas in individual cases of pancreatic neuroendocrine tumors it shows a normal expression pattern without abnormalities (178), a feature similar to that of ADAM9. Based on this specific expression characteristic, WNT7A may serve as a potential antigen candidate for the development of vaccines for PDAC patients. While significant progress has been made in studying WNT7A in tumor proliferation and invasion, related research in the field of pancreatic cancer remains relatively limited. At the immunogenic level, inhibition of WNT7A expression can significantly upregulate the expression level of MHC class I molecules in tumor cells, thereby promoting the tumor infiltration of CD8^+^ T cells and enhancing their cytotoxic function (179), and another study has also confirmed the same negative correlation between the two (180). From the perspective of immunogenicity, WNT7A is not suitable as a vaccine candidate antigen; although this molecule can participate in multiple signaling pathways to regulate the oncogenesis and progression of tumors, the feasibility of its use as a potential antigen for tumor vaccines remains to be debated.

### 5.5 AGPS

AGPS is a rate-limiting enzyme in ether lipid biosynthesis and has been implicated in tumor aggressiveness through metabolic rewiring (181–183). Mechanistically, AGPS may promote pro-tumor ether lipid production and alter arachidonic acid flux (183), while related lipid metabolic enzymes (for example, MAGL) can converge on similar phenotypes (184). Emerging work further suggests that AGPS-regulated ether lipid species may intersect with redox control and ferroptosis susceptibility, providing a potential mechanistic bridge between metabolic vulnerability and immunotherapy (185, 186). However, PDAC-specific data remain limited, and the immunological tractability of AGPS as a vaccine antigen is currently unclear. As such, AGPS is best viewed as an exploratory metabolic antigen whose relevance may lie in combination strategies linking vaccination with ferroptosis-oriented therapeutic modulation.

## 6 Summary of antigen stratification and insights for mRNA vaccine design

Collectively, the 16 candidate antigens reviewed in this study, although derived from systematic screening efforts, differ substantially in the strength of supporting evidence, immunological maturity, and translational readiness. Treating all candidates equivalently risks obscuring their true value for vaccine development and may misguide downstream experimental and clinical strategies. Explicit stratification of these antigens is therefore essential for rational mRNA vaccine design in pancreatic cancer.

### 6.1 Stratification framework and translational priorities

Based on a comprehensive assessment of tumor-specific expression, functional relevance, and immunogenicity evidence, these 16 antigens can be clearly divided into three categories.

High-confidence antigens (MET, SCP-1, GAGE, PAK2, and TPX2) exhibit consistent overexpression in pancreatic cancer and play established roles in tumor progression or immune activation. Several possess defined T-cell epitopes or direct evidence of immune responsiveness, conferring advantages in both safety and functional relevance. These antigens should be prioritized as core targets for pancreatic cancer mRNA vaccine development.

Functionally associated antigens (ADAM9, EFNB2, KDM5A, ANO6, RAB5A, and TMOD3) represent key regulatory nodes within oncogenic signaling, metabolic reprogramming, or regulated cell death pathways. Although direct immunogenicity evidence remains limited, their value lies in providing functional leverage for vaccine design, particularly as components of multi-antigen or pathway-synergistic strategies rather than standalone targets.

Exploratory antigens (OSBPL9, NRAS, CHMP2B, WNT7A, and AGPS) have so far mainly been proposed based on bioinformatics or correlative analyses, and their immunological significance in pancreatic cancer remains unclear. These antigens should not be regarded as direct candidates for clinical translation at this stage, but are better suited as hypothesis-generating targets under new screening strategies, providing clues for future mechanistic studies.

### 6.2 The mTOR pathway in tumor vaccine antigen screening

The mTOR pathway is widely involved in multiple physiological processes such as nutrient metabolism (187, 188), transcriptional regulation (189), cell growth and proliferation in the organism, and relevant research has become a research hotspot in this field. This pathway can regulate gene expression programs and mediate key biological processes including mRNA translation through various molecular mechanisms (190, 191); moreover, existing studies have confirmed that aberrant activation of mTOR can significantly promote tumorigenesis and metastasis, and this pathway is also able to participate in the differentiation and development of CD8^+^ T cells, thereby enhancing the body’s anti-tumor immune response (192). Immunogenicity is a core evaluation index for the screening of candidate antigens for mRNA vaccines, and the mTOR pathway can regulate processes closely associated with immunogenicity such as immune antigen presentation and mRNA translation. Therefore, incorporating the regulatory characteristics of the mTOR pathway into the potential screening and evaluation system for vaccine candidate antigens can provide an important reference for the screening of high-efficiency antigens. Relevant studies on oocyte development have shown that activation of the PI3K-AKT-mTOR pathway can regulate the mRNA translation process, thereby participating in the regulation of oocyte development (193). In view of this, investigating the association mechanism between potential vaccine antigens and the mTOR pathway, and clarifying their roles via the regulation of mRNA translation, can provide an important theoretical basis for the screening of vaccine candidate antigens. SCP-1 can significantly inhibit the expression of related proteins in the PI3K/Akt/mTOR signaling pathway, thereby mediating autophagy and apoptosis in tumor cells and enhancing the body’s anti-tumor activity (194). In addition, melatonin can regulate the AMPK pathway and participate in the cellular autophagy process by upregulating the expression of PAK2 and inhibiting mTOR phosphorylation, which provides a new research perspective for exploring whether this regulatory mechanism is involved in the oncogenesis and progression of pancreatic cancer (195). At present, relevant research in this field remains relatively scarce; therefore, in-depth investigation of the correlation between potential vaccine antigen targets and the mTOR pathway will provide critical experimental evidence and novel research directions for the development and preparation of tumor vaccines.

### 6.3 HLA and epitope presentation of candidate antigens

Human leukocyte antigen (HLA) molecules are core mediators of antigen presentation. They bind and present short peptide fragments derived from antigens on the cell surface for recognition by CD8^+^ cytotoxic T cells and CD4^+^ helper T cells, thereby initiating specific anti-tumor immune responses. The translational value of mRNA vaccines highly depends on whether candidate antigens can generate immunodominant epitopes that bind to prevalent HLA alleles, so as to induce efficient and stable anti-tumor T cell responses in a wide population.

Current studies have confirmed clear HLA restriction patterns for several candidate antigens. Processed peptides from SCP−1 can elicit specific CD4^+^ T cell responses restricted by HLA−DRB1*1401, further supporting its potential as a vaccine antigen target (60). GAGE family antigens can be effectively presented by HLA class I molecules. Among them, the antigenic peptide YRPRRY encoded by GAGE−1 has been verified to be presented by HLA−Cw6 on the surface of melanoma cells (61, 196). PAK2 shows high binding affinity to HLA−A2, suggesting efficient presentation via this HLA allele (80). c−MET can be robustly presented by HLA−A*0201 (197), and TPX2 can also be presented by the same allele, as its peptide fragments exhibit high binding rates to HLA−A*0201, laying the foundation for activating specific immunity in dominant populations (198).

At present, HLA restriction of most candidate antigens lacks systematic validation, especially with insufficient evidence for epitope presentation and immune activation in the context of pancreatic cancer. Further studies are urgently needed to identify the HLA restriction profiles of these antigens, clarify their epitope presentation characteristics and population coverage, and further enhance their translational value as candidate antigens for pancreatic cancer mRNA vaccines.

### 6.4 Implications for antigen selection strategies

MET and ADAM9 are both highly expressed in pancreatic cancer tissues and show broader population coverage compared with other candidate antigens. Among them, MET is highly expressed not only in pancreatic cancer but also in a variety of other malignant tumors, and its favorable immunogenicity endows it with great potential as a broad-spectrum tumor vaccine antigen. Although current research on MET in pancreatic cancer remains limited, its comprehensive advantages are prominent, making it the highest-priority candidate antigen for further in-depth investigation.SCP-1 and GAGE belong to the cancer-testis (CT) antigen family and also possess excellent immunogenicity. Among them, peptides derived from SCP-1 have been used in the development of peptide vaccines and entered clinical trials, indicating clear translational value. However, a common limitation of both antigens is that their expression is not specific to pancreatic cancer, with certain expression observed in normal tissues, making it difficult to achieve precise tumor targeting and thereby limiting their application prospects. PAK2 and TPX2 have been confirmed to be highly expressed in pancreatic cancer with definite immunogenicity. Nevertheless, relevant research is still insufficient to support their superiority over MET, SCP-1, and GAGE as more advantageous candidate antigens. With the continuous progress of clinical translational research and trials in the future, the application potential of both antigens is expected to be further explored and validated.

The hierarchical results above suggest that the strategy for antigen selection in pancreatic cancer mRNA vaccines is undergoing an important shift. The traditional approach of prioritizing “high expression” is no longer sufficient to meet the demands of vaccine design in the complex tumor immune environment. In contrast, a comprehensive evaluation system that takes into account tumor specificity, functional dependency, and immune activation potential is more conducive to identifying antigens with true translational value.

In addition, as a typical “immunologically cold tumor,” pancreatic cancer often fails to elicit a sufficiently strong immune response from single-antigen vaccines. The functionally associated antigens summarized in this article suggest that future mRNA vaccine designs could consider combining multiple antigens or coupling functional pathways. This approach would not only activate specific immune cytotoxicity but also weaken the tumor cells’ dependence on certain survival pathways, thereby amplifying the overall anti-tumor effect.

### 6.5 Translational barriers for mRNA vaccine development in pancreatic cancer

Despite encouraging advances, several translational barriers continue to limit the clinical efficacy of mRNA vaccines in pancreatic cancer. First, delivery efficiency remains a critical challenge. Although lipid nanoparticle (LNP) systems have significantly improved mRNA stability and cellular uptake, their biodistribution, endosomal escape efficiency, and targeting specificity in pancreatic ductal adenocarcinoma (PDAC) are still suboptimal, particularly due to the dense stromal architecture and abnormal vasculature (29, 31).

Second, the highly immunosuppressive tumor microenvironment (TME) further compromises vaccine efficacy. PDAC is characterized by abundant regulatory T cells (Tregs), myeloid-derived suppressor cells, and tumor-associated macrophages, along with extensive desmoplasia, which together create both biochemical and physical barriers to immune cell infiltration and function (32, 33).

Third, HLA restriction represents an additional constraint on clinical applicability. The immunogenicity of candidate antigens depends on their ability to generate peptides that bind specific HLA alleles, thereby restricting their effectiveness to subsets of patients (199).

### 6.6 Directions for multi-antigen and combination therapy strategies

The combined antigen strategy aims to simultaneously deliver multiple tumor-associated antigens to achieve synergistic immune activation, thereby overcoming the limitations of single-antigen approaches, including restricted patient coverage and insufficient immune response induction. This strategy is particularly relevant for pancreatic cancer, which is characterized by pronounced tumor heterogeneity and a high propensity for immune escape.

Based on the antigen stratification framework proposed in this study, an optimized design may involve combining high-confidence antigens with functionally associated candidates to achieve both robust immune activation and broader biological targeting. For example, combinations centered on MET with additional antigens such as PAK2 and TPX2 may enhance epitope diversity while targeting proliferation and cell cycle–related pathways. Alternatively, integrating MET with ADAM9 and KDM5A may enable simultaneous modulation of tumor progression and the tumor microenvironment.

In terms of delivery, multi-antigen strategies may be implemented by co-encapsulating multiple mRNA constructs within a single lipid nanoparticle (LNP) formulation or by combining separately encapsulated mRNAs at optimized ratios, enabling synchronized antigen expression *in vivo (*192). Emerging studies in other tumor types, such as breast cancer, have demonstrated the feasibility of such multi-antigen mRNA vaccine designs, providing a valuable reference for pancreatic cancer (193).

## 7 Conclusion and outlook

mRNA vaccine development for pancreatic cancer is transitioning from proof-of-concept studies toward strategic optimization, with antigen selection emerging as the central determinant of translational success. By systematically reviewing and stratifying 16 candidate antigens, this study highlights the marked heterogeneity in evidentiary support and clinical potential among proposed targets. Our analysis indicates that transcriptional overexpression alone is insufficient for antigen prioritization; instead, effective vaccine targets should integrate tumor specificity, functional relevance, immune context, and immunogenic potential. This stratification framework provides a rational basis for multi-antigen vaccine design. Rather than relying on single highly expressed antigens, combinatorial strategies may offer improved efficacy by addressing tumor heterogeneity and immune escape. For example, MET-centered combinations incorporating PAK2 and TPX2 may enhance epitope diversity while targeting proliferation-related pathways, whereas integration with functionally associated antigens such as ADAM9 and KDM5A may further modulate tumor progression and the immunosuppressive microenvironment. These designs represent hypothesis-driven strategies for future validation. Significant challenges remain, including the need for rigorous *in vivo* and *in vitro* validation of antigen immunogenicity and safety, as well as overcoming immune suppression within the pancreatic tumor microenvironment. Stratification-guided multi-antigen vaccine design, combined with complementary immunotherapeutic or targeted approaches, represents a promising avenue to address these barriers.

Overall, this review not only synthesizes current knowledge on pancreatic cancer mRNA vaccine antigens but also proposes a conceptual framework for antigen prioritization, combination design, and translational progression. With continued advances in multi-omics technologies and tumor immunology, pancreatic cancer mRNA vaccines are expected to advance from computational screening toward functional validation and clinical exploration, offering new therapeutic possibilities for this highly lethal disease.

## Data Availability

The original contributions presented in the study are included in the article/supplementary material. Further inquiries can be directed to the corresponding authors.
